# DEWAX Transcription Factor Is Involved in Resistance to *Botrytis cinerea* in *Arabidopsis thaliana* and *Camelina sativa*

**DOI:** 10.3389/fpls.2017.01210

**Published:** 2017-07-11

**Authors:** Seulgi Ju, Young Sam Go, Hyo Ju Choi, Jeong Mee Park, Mi Chung Suh

**Affiliations:** ^1^Department of Bioenergy Science and Technology, Chonnam National University Gwangju, South Korea; ^2^Plant Systems Engineering Research Center, Korea Research Institute of Bioscience and Biotechnology Deajeon, South Korea; ^3^Department of Biosystems and Bioengineering, KRIBB School of Biotechnology, Korea University of Science and Technology Daejeon, South Korea

**Keywords:** AP2/ERF-type transcription factor, Arabidopsis thaliana, Botrytis cinerea, Camelina sativa, DEWAX, transcriptional regulation

## Abstract

The cuticle of land plants is the first physical barrier to protect their aerial parts from biotic and abiotic stresses. DEWAX, an AP2/ERF-type transcription factor, negatively regulates cuticular wax biosynthesis. In this study, we investigated the resistance to *Botrytis cinerea* in *Arabidopsis thaliana* and *Camelina sativa* overexpressing *DEWAX* and in *Arabidopsis dewax* mutant. Compared to wild type (WT) leaves, *Arabidopsis DEWAX* OX and *dewax* leaves were more and less permeable to toluidine blue dye, respectively. The ROS levels increased in *DEWAX* OX leaves, but decreased in *dewax* relative to WT leaves. Compared to WT, *DEWAX* OX was more resistant, while *dewax* was more sensitive to *B. cinerea*; however, defense responses to *Pseudomonas syringae* pv. tomato DC3000:GFP were inversely modulated. Microarray and RT-PCR analyses indicated that the expression of defense-related genes was upregulated in *DEWAX* OX, but downregulated in *dewax* relative to WT. Transactivation assay showed that DEWAX upregulated the expression of *PDF1.2a, IGMT1*, and *PRX37*. Chromatin immunoprecipitation assay revealed that DEWAX directly interacts with the GCC-box motifs of PDF1.2a promoter. In addition, ectopic expression of *DEWAX* increased the tolerance to *B. cinerea* in *C. sativa.* Taken together, we suggest that increased ROS accumulation and *DEWAX*-mediated upregulation of defense-related genes are closely associated with enhanced resistance to *B. cinerea* in *Arabidopsis* and *C. sativa*.

## Introduction

The hydrophobic cuticle covering the aboveground parts of land plants forms a physical and chemical barrier to infection by bacterial and fungal pathogens, as well as drought stress (Nawrath, 2006; Pollard et al., 2008; Beisson et al., 2012; Li-Beisson et al., 2013; Yeats and Rose, 2013). The cuticle is composed of a cutin polyester layer, an intracuticular wax layer embedded in the cutin layer, and an outermost epicuticular wax layer (Bernard and Joubes, 2013; Yeats and Rose, 2013). The structural polymer cutin is cross-linked *via* ester bonds among glycerols and C16 and C18 polyhydroxy, epoxy, ω-hydroxy, and α,ω-dicarboxylic fatty acid components (Pollard et al., 2008; Beisson et al., 2012). Cuticular wax is a mixture of very-long-chain fatty acids and their derivatives, such as alkanes, aldehydes, ketones, primary and secondary alcohols, and wax esters (Kunst and Samuels, 2009; Li-Beisson et al., 2013; Lee and Suh, 2015). The cutin and wax components are synthesized from C16 and C18 fatty acid precursors produced in the plastids by the endoplasmic reticulum (ER)-localized enzymes and exported to the extracellular space through the cytoplasmic ATP-binding cassette transporters (Pighin et al., 2004; Bird et al., 2007; Panikashvili et al., 2007, 2011; Kunst and Samuels, 2009; McFarlane et al., 2010; Bessire et al., 2011; Bernard and Joubes, 2013; Li-Beisson et al., 2013; Lee and Suh, 2015).

When pathogenic fungi come in contact with a host plant, they first secrete cutinase or lipase to break down the cutin layer for penetration into the plant body (Kolattukudy, 1985; Kolattukudy et al., 1995). Degraded cutin is recognized by pathogen and promotes disease development, whereas as a counterprogram, it is recognized by the plant immune system and acts as a signal transmitter to induce an immune response (Woloshuk and Kolattukudy, 1986; Schweizer et al., 1996). Overexpression of a fungal cutinase in *Arabidopsis* plants enhanced solute permeability and led to increased resistance to the necrotrophic fungus *Botrytis cinerea* (Chassot et al., 2007). Several *Arabidopsis* mutants with increased cuticular permeability, including *lacerate* (*lcr*), *bodyguard* (*bdg*), *long-chain acyl-CoA synthetase-2* (*lacs-2*) and *-3, fiddlehead* (*fdh*), *permeable cuticle1* (*fec1*), and *myeloblast transcription factor 96* (*myb96*), also display enhanced resistance to *B. cinerea* (Yephremov et al., 1999; Wellesen et al., 2001; Kurdyukov et al., 2006; Bessire et al., 2007, 2011; Chassot et al., 2007; Voisin et al., 2009; Seo et al., 2011). However, some *Arabidopsis* mutants with defective cuticle, such as *glycerol-3-phosphate acyltransferase4 gpat8* (*gpat4 gpat8*), *acyl-CoA binding protein1* (*acbp1*), and *glycosylphosphatidyl-anchored lipid transfer protein1* (*ltpg1*), are more susceptible to fungal pathogens, including *B. cinerea* and *Alternaria brassicicola* (Li et al., 2007; Lee et al., 2009; Xue et al., 2014). Xia et al. (2009) showed that an intact cuticle layer is involved in the systemically acquired resistance (SAR) response, and cuticle-mediated SAR is independent of salicylic acid- or jasmonic acid-mediated defense signaling. Disruption of *acbp3, 4*, and *6* increased the cuticle permeability and susceptibility to bacterial and fungal pathogens due to defective SAR signal generation (Xia et al., 2012). Therefore, it is important to investigate the roles of the cuticle in plant immunity.

Cuticular wax biosynthesis is controlled by various environmental stress conditions, and several transcription factors involved in its regulation have been reported (Jenks et al., 2001; Cameron et al., 2006; Shepherd and Griffiths, 2006; Raffaele et al., 2007; Kosma et al., 2009; Seo et al., 2011; Lee and Suh, 2013, 2015; Go et al., 2014; Kim et al., 2017). Among them, MYB30 modulates hypersensitive cell death-related lipid signaling by the upregulation of very-long-chain fatty acid synthesis (Raffaele et al., 2007). MYB96 activation-tagging lines with increased total wax load showed resistance to the virulent pathogen, *Pseudomonas syringae* pv. tomato DC3000, by inducing salicylic acid biosynthesis (Seo and Park, 2010). The APETALA2/Ethylene-Responsive Factor (AP2/ERF)-type transcription factor DEWAX represses cuticular wax biosynthesis *via* downregulation of the cuticular wax biosynthetic genes *CER1, LACS2, ACLA2*, and *ECR* (Go et al., 2014). However, defense responses of *DEWAX* overexpression (OX) lines, which display a permeable cuticle, against pathogen attacks have not yet been reported.

*Camelina sativa*, which belongs to the *Brassicaceae* family, has recently gained increasing interest for its potential non-food usages, including in cosmetics, lubricants, and as biofuels (Moser, 2010; Waraich et al., 2013). *C. sativa* has a short life cycle (100–120 days), and can be grown on marginal agricultural lands owing to its characteristics, such as tolerance to cold stress, relatively good growth under nutrient-poor soil conditions, and high levels of polyunsaturated fatty acids in the seed oils (Putnam et al., 1993; Zubr, 1997; Enjalbert et al., 2013). To date, transgenic *C. sativa* plants with high seed-oil content or enhanced drought resistance have been developed *via Agrobacterium*-mediated transformation (Liu et al., 2012; Lee et al., 2014; An and Suh, 2015; Kim et al., 2016). However, only few reports on genetically improved *C. sativa* with enhanced tolerance to pathogens are available (Zakharchenko et al., 2013).

In this study, we observed that *DEWAX* OX plants were more resistant to the fungal pathogen, *B. cinerea*, but were more susceptible to the bacterial pathogen, *Pto DC3000* compared with wild type (WT). To understand the molecular mechanisms underlying the defense responses of *Arabidopsis* and *C. sativa* plants overexpressing *DEWAX* to *B. cinerea*, further analyses were carried out. Finally, we revealed that DEWAX plays as a transcriptional activator that upregulates the expression of the defense-related genes such as *PDF1.2a, IGMT1*, and *PRX37 via* binding to their gene promoters. The results suggest that *DEWAX*-mediated upregulation of defense-related genes in addition to an increase in ROS levels is important in the enhanced tolerance to *B. cinerea* in *Arabidopsis* and *C. sativa* overexpressing *DEWAX*.

## Materials and Methods

### Plant Materials and Growth Conditions

*DEWAX* overexpression lines (OX1 and OX2) and *dewax* knockout mutant were described in Go et al. (2014). *A. thaliana* (ecotype Columbia-0) seeds were surface-sterilized with 75% (v/v) ethanol and 0.05% (v/v) Triton X-100, washed with 100% ethanol, dried on filter paper, and germinated on half MS (Murashuge and Skoog, 1962) medium containing 1% sucrose and 0.6% phytoagar (Duchefa), which was adjusted to pH 5.7 using KOH. Seven-day-old seedlings were transplanted to mixed soil (soil/vermiculite/perlite, 3:2:1, v/v/v) and grown in a growth room under long-day conditions (16-h-light and 8-h-dark cycle) at 24 ± 2°C. *C. sativa* ‘CAME’ was grown on mixed soil (soil/vermiculite/perlite, 3:2:1, v/v/v) at 25 ± 3°C under a 16-h-light/8-h-dark cycle (Lee et al., 2014).

### RNA Isolation and Quantitative RT-PCR Analysis

Total RNA was isolated from 4-week-old Col-0, *dewax, DEWAX* lines (OX1 and OX2), and 6-week-old transgenic *C. sativa* lines (T_1_ generation) using the NucleoSpin RNA Plant Extraction Kit (Macherey-Nagel) according to the instructions of the manufacturer. The total RNA was reverse-transcribed with GoScript^TM^ Reverse Transcription System (Promega), and the cDNA was used in RT-PCR analysis. RT-PCR was performed using the Access Quick^TM^ RT-PCR system (Promega, Madison, WI, United States) with gene-specific primers (Supplementary Table S1). *Arabidopsis EIF4* (At3g13920) and *C. sativa ACTIN11* genes (Hutcheon et al., 2010) were used in RT-PCR analysis as a control for cDNA quality and quantity. Quantitative RT-PCR (qRT-PCR) was executed using SYBR^®^ FAST Universal 2x qPCR Master Mix (KAPA Biosystems, Wilmington, MA, United States) in a final volume of 20 μL. PCR was performed according to the manufacturer’s protocols using gene specific primers described in Supplementary Table S1. *PP2A* (At1g13320) and *CsACT11* genes were used to determine the RNA quality and quantity.

### Toluidine Blue Staining

Four-week-old *Arabidopsis* rosette leaves were stained at room temperature for 8 min without shaking using freshly prepared staining solution containing 0.05% toluidine blue and 0.01% Tween-20. After staining, leaves were rinsed five times with distilled water and photographed.

### ROS Assay

For *in situ* detection of hydrogen peroxide, *Arabidopsis* leaves were immersed in 10 mM MES (pH 6.5) containing 0.1% 3,3′-diaminobenzidine tetrahydrochloride (DAB, Sigma-Aldrich), gently vacuum-infiltrated, and incubated for 30 min as described in Piisilä et al. (2015). After staining, the leaves were de-stained by boiling in ethanol-lactophenol (2:1) for 5 min, washed twice with 50% ethanol, rinsed once with distilled water, and then photographed. For fluorometric measurements of ROS levels, *Arabidopsis* leaves were incubated in 10% MS salt solution containing 20 μM 2′,7′-dichlorofluorescein diacetate (H_2_DCFDA, Sigma-Aldrich) and 0.1% Tween-20 in the dark for 30 min. DCF fluorescence in aliquots taken from the incubation solution was measured using a Synergy H1 Hybrid Multi-Mode Microplate Reader (BioTek) at 488 and 525 nm. Leaf fresh weights were used to normalize the values (Umbach et al., 2012). For measurement of hydrogen peroxide (H_2_O_2_) content, 5-week-old leaves (200 mg) were incubated in 1.5 mL of 5 mM morpholineethanesulfonic acid (MES)-KOH buffer (pH 6.7) for 7 h (Horemans et al., 2007). Peroxidase reaction was started by adding 3,5-dichloro-2-hydroxyvenxenesulfonic acid (DCHBS), 4-aminoantipyrine (4-AAP), and peroxidase based on the manufacturer’s instructions (Megazyme) and the reaction mixture was incubated for 1 h at 25°C. Absorbance for the quinoneimine dye was spectrophotometrically measured using a Synergy H1 Hybrid Multi-Mode Microplate Reader (BioTek) at 520 nm. H_2_O_2_ content was estimated using a standard curve prepared similarly with 0–3 μM H_2_O_2_.

### Pathogen Inoculation

*Botrytis cinerea* strain B05.10 was kindly provided by Dr. Mengiste (Purdue University, United States) and was cultured on 2× V8 tomato agar (36% V8 juice, 0.2% CaCO_3_, and 2% Bacto-agar). Harvesting and inoculation of conidia were carried out according to Veronese et al. (2006). Detached *Arabidopsis* leaves were laid out on 3MM paper with water and inoculated with 5-μl droplets of conidial suspension (5 × 10^5^ spores/mL), kept under a sealed transparent cover to maintain high humidity and transferred to a growth chamber (21 ± 2°C, 16 h light/8 h dark). *C. sativa* plants were sprayed with *B. cinerea* conidial suspension (2 × 10^5^ spores/mL). After inoculation, the plants were transferred to growth chambers (21 ± 2°C, 16 h light/8 h dark) and covered with a sealed clear lid to maintain low light intensity and high humidity. Quantification of *B. cinerea* biomass in *C. sativa* was carried out according to Gachon and Saindrenan (2004).

A *Pto* DC3000 strain harboring pDSK-GFPuv (*Pto* DC3000:GFP, Wang et al., 2007) was used for dip inoculation (Katagiri et al., 2002). Briefly, 3-week-old *Arabidopsis* plants grown in meshed pots were dipped in 50 ml of bacterial suspensions (optical density at 600 nm of 0.1) containing 0.05% Silwet L-77 (Lehle Seeds, Round Rock, TX, United States) for few seconds. After inoculation, the plants were covered with a clear lid and transferred to a growth room (23 ± 2°C, 16 h light/8 h dark). Inoculated plants were photographed under ultraviolet light to determine *Pto*DC3000:GFP proliferation using GFP signals and bacterial populations were measured by serial dilution assays (Katagiri et al., 2002). Colony forming unit (CFU) was normalized as CFU/mg using total weights of the inoculated leaves.

### Transactivation Assay in Yeast

For the transcactivation assay in yeast, *DEWAX-F* (603 bp for full-length), *DEWAX-N* (312 bp for N-terminal region), and *DEWAX-C* (291 bp for C-terminal region) DNA fragments were amplified *via* PCR using *DEWAX* cDNA and *DEWAX*-specific primers (Supplementary Table S1). *Sma*I and *Sac*I-digested PCR fragments were cloned into the pGBKT7 vector (BD Biosciences Clontech). The recombinant vector (pAtDEWAX-F, pAtDEWAX-N, and pAtDEWAX-C) were transformed into yeast strain Y190 (MATa, HIS3, lacZ, trp1, leu2, cyhr2) according to the manufacturer’s instructions (BD Biosciences Clontech). Transformants were selected on selective medium (SD-Trp) supplemented with 25 mM 3-amino-1, 2, 4-aminotriazole (3-AT). Transformants were cultured on selective medium (SD-Trp-His) for 1 day at 30°C and filter-lift assays were performed for 16 h at 37°C, as described in Breeden and Nasmyth (1985).

### Transcriptional Activation Assay

To generate the reporter constructs, DNA fragments corresponding to the PDF1.2a (∼1.3 kb), IGMT (∼1.5 kb), PRX37 (∼1.2 kb) promoter regions were amplified using gene-specific primers and cloned into the *Sal*I and *Spe*I sites of the GAL4-LUC binary vector. The recombinant pPZP212 vector (Hajdukiewicz et al., 1994) harboring *Arabidopsis DEWAX* driven by the CaMV35S promoter, reported by Go et al. (2014), was used as an effector construct. Protoplast isolation was carried out according to Yoo et al. (2007).

The reporter and effector constructs were isolated using a Qiagen Plasmid Midi Kit and co-introduced into tobacco protoplasts (Miura et al., 2007). After incubation of the transfected tobacco protoplasts in the dark at 23°C for 16 h, the protoplasts were subjected to enzymatic assays to quantify luciferase and β-glucuronidase activities using a dual-luciferase assay system (Promega). Luciferase gene expression was normalized to that of the β-glucuronidase gene.

### ChIP Assay

Chromatin immunoprecipitation (ChIP) assay was performed using 35S:MYC-DEWAX transgenic plants, which were previously reported (Go et al., 2014). Genomic DNA was isolated from 10-day-old 35S:MYC-DEWAX transgenic seedlings and immune-precipitated with an anti-myc antibody (Santa Cruz Biotechnology). Quantitative RT-PCR was performed as described previously (Gendrel et al., 2005). *Arabidopsis actin7* (At5g09810) was used to control cDNA quantity and quality.

### Construction of a Binary Vector and Transformation of *C. sativa*

To generate transgenic *C. sativa* plants overexpressing *Arabidopsis DEWAX*, the full-length *DEWAX* gene was amplified from cDNA converted from RNA isolated from 3-week-old leaves with DEWAX F1 and DEWAX R1 primers (Supplementary Table S1). The PCR product was digested with *Sac*1 and *Sma*1 and cloned into the binary vector pBA002. The binary vectors were transformed into *Agrobacterium* strain GV3101 by the freeze-thaw method (An, 1987).

*Agrobacterium* harboring the 35S:MYC-DEWAX construct (Go et al., 2014) was inoculated in YEP medium (containing 50 μg/mL rifampicin and 50 μg/mL spectinomycin) and cultured at 30°C overnight with shaking. The culture was centrifuged at 3,000 rpm for 20 min and the pellet was resuspended in transformation solution (5% sucrose and 0.05% Silwet L-77) at an OD_600_ of 0.8. Five- to six-week-old *C. sativa* plants were transformed with *Agrobacterium* by a modified floral dip method (Lu and Kang, 2008; Liu et al., 2012). T_1_ transgenic seeds were sowed on mixed soil and selected by spraying of 0.03% (v/v) BASTA herbicide.

### Genomic DNA Isolation and PCR Analysis

Genomic DNA was isolated from leaves of 3-week old non-transgenic and transgenic *C. sativa* plants (T_1_ generation) using extraction buffer (200 mM Tris-HCl, pH 7.5, 250 mM NaCl, 25 mM EDTA, and 0.5% sodium dodecyl sulfate) and subjected to genomic DNA PCR analysis with CaMV35S promoter F and DEWAX CR1 primers (Supplementary Table S1). Thermal cycles included 32 cycles of denaturation at 94°C for 30 s, annealing at 60°C for 30 s, and elongation at 72°C for 60 s.

## Results

### Overexpression of *DEWAX* Increased Cuticle Permeability and ROS Accumulation in *Arabidopsis*

**FIGURE 1:**
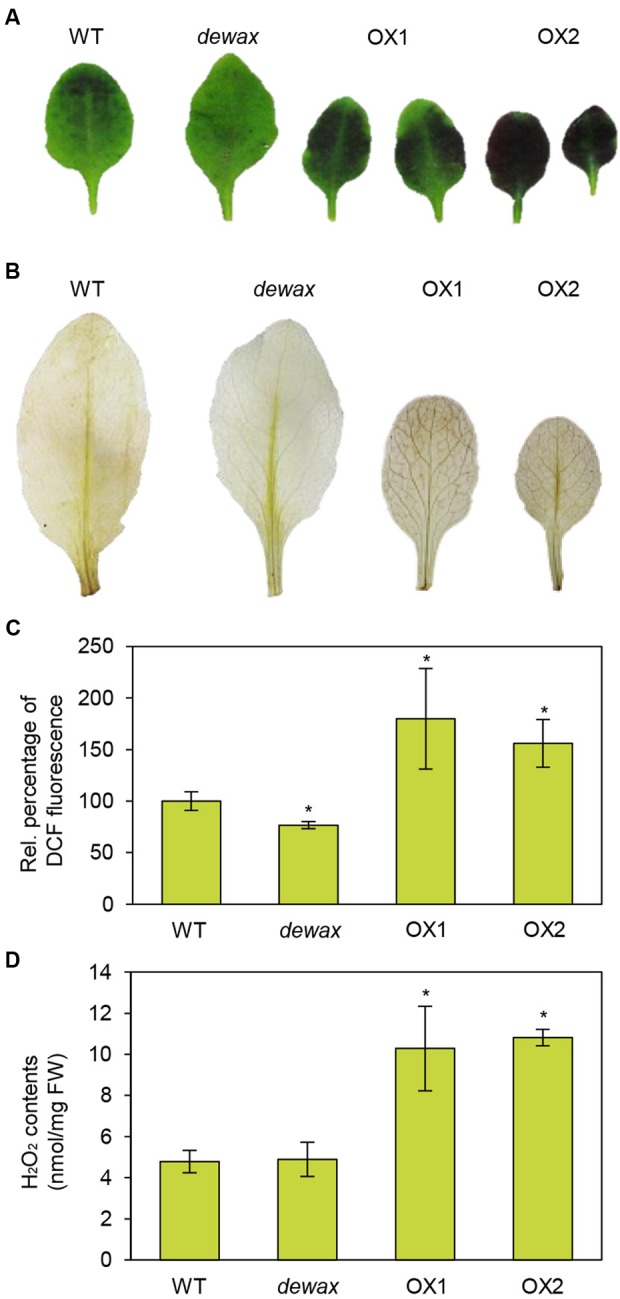
Overexpression of *DEWAX* increased cuticle permeability and ROS accumulation in *Arabidopsis*. **(A)** Cuticle permeability analysis. Leaves of WT, *dewax*, and *DEWAX* overexpression lines were stained with 0.05% toluidine blue for 8 min. **(B)** *In situ* detection of hydrogen peroxide. Leaves of WT, *dewax*, and *DEWAX* overexpression lines were incubated in 10 mM MES (pH 6.5) containing 0.1% DAB for 30 min, de-stained by boiling in ethanol-lactophenol (2:1) for 5 min, washed with 50% ethanol, rinsed with distilled water, and then photographed. **(C)** Measurements of ROS levels. Leaves of WT, *dewax*, and *DEWAX* overexpression lines were incubated in 10% MS salt solution containing 20 μM H_2_DCFDA and 0.1% Tween-20 for 30 min. DCF fluorescence in aliquots taken from the extracts was measured at 488 and 525 nm. Leaf fresh weights were used to normalize the values. Data were statistically analyzed using Student’s *t*-test (^∗^*P* < 0.05). Error bars indicate ± SD from triplicate experiments. **(D)** Measurements of hydrogen peroxide content. Five-week-old WT, *dewax*, OX1, and OX2 leaves (200 mg) were incubated in 1.5 mL of 5 mM MES buffer (pH 6.7) for 7 h. Peroxidase reaction was started by adding DCHBS, 4-AAP, and peroxidase (Megazyme) and the reaction mixture was incubated for 1 h at 25°C. Absorbance for the quinoneimine dye was spectrophotometrically measured at 520 nm. H_2_O_2_ content was estimated using a standard curve. Data were statistically analyzed using Student’s *t*-test (^∗^*P* < 0.01). Error bars indicate ± SD from triplicate experiments.

To investigate the cuticle permeability of WT, *dewax*, and two *DEWAX* OX lines, rosette leaves of 4-week-old plants were stained with 0.05% toluidine blue. As shown in **Figure 1A**, compared to WT, *Arabidopsis DEWAX* OX and *dewax* leaves were more and less permeable to toluidine blue dye, respectively. Based on the previous reports that *Arabidopsis bdg* and *lacs2* mutants with a permeable cuticle showed increased ROS levels (L’Haridon et al., 2011), ROS assay was performed in the leaves of WT, *dewax*, and *DEWAX* OX lines. Both *in situ* assay of hydrogen peroxide and fluorometric measurements of ROS levels showed that the levels of ROS were lower in *dewax* leaves, but higher in *DEWAX* OX1 and OX2 leaves than in WT leaves (**Figures 1B,C**). When hydrogen peroxide content was spectrophotometrically measured after leaves of WT, *dewax*, and *DEWAX* OX lines were preadapted in incubation buffer, the levels of hydrogen peroxide were approximately twofold higher in *DEWAX* OX1 and OX2 leaves than in WT leaves, but no remarkable differences were detected between WT and *dewax* leaves (**Figure 1D**).

### Overexpression of *DEWAX* Enhanced Resistance, While Disruption of *DEWAX* Increased Susceptibility to *B. cinerea*

**FIGURE 2:**
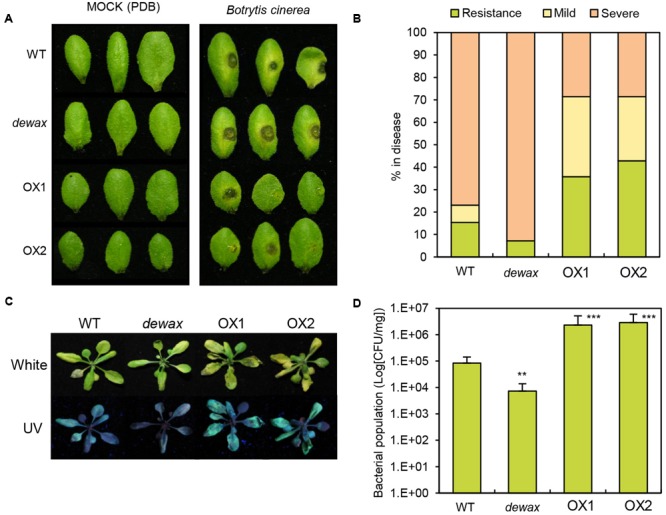
Overexpression of *DEWAX* confers tolerance to *Botrytis cinerea* and susceptibility to *Pto* DC3000:GFP in transgenic *Arabidopsis*. **(A)** Macroscopic symptoms on leaves of WT, *dewax*, and *DEWAX* overexpression lines at 6 days after inoculation with potato dextrose broth (Mock) or *B. cinerea*. **(B)** Disease symptom scores at 6 days after inoculation with *B. cinerea* (0 = non-infection, 1 = lesions at the point of inoculation, 2 = larger lesions surrounded by extensive chlorosis). **(C)** Disease phenotypes of WT, *dewax* and *DEWAX* overexpression lines after dip-inoculation with *Pto* DC3000:GFP bacteria. Photographs were taken 3 dpi under white and UV lights. **(D)** Bacterial populations of *Pto* DC3000:GFP in WT, *dewax* and *DEWAX* overexpression lines were measured at 3 dpi. Colony forming unit (CFU) was normalized as CFU/mg using total weights of the inoculated leaves. The results are representative of three independent experiments. Error bars indicate SE. Asterisks indicate a statistically significant difference compared with wild type (WT) using Student’s *t*-test (^∗∗^*P* < 0.01, ^∗∗∗^*P* < 0.001).

To investigate the response of WT, *dewax*, and two *DEWAX* OX lines to the necrotrophic pathogen *B. cinerea* and the hemibiotrophic bacterial pathogen *Pto* DC3000, first, we examined visible disease symptoms 72 h after inoculation of *B. cinerea* on the adaxial surface of the leaves. In a spot inoculation assay, significantly larger lesions were observed on the leaves of *dewax* mutant, while smaller lesions were formed on *DEWAX* OX as compared with WT leaves (**Figures 2A,B**), indicating that *DEWAX* OX shows enhanced resistance, and *dewax* enhanced susceptibility, to *B. cinerea*. On the other hand, compared to WT, *DEWAX* OX was more susceptible to *Pto* DC3000:GFP, while *dewax* was more resistant to this pathogen (**Figures 2C,D**). These results indicate that DEWAX is involved in the disease resistance responses of *Arabidopsis* and plays a positive role in the defense to fungal pathogens, but a negative role in the defense to bacterial pathogens.

### Expression of DEWAX-Regulated Genes in *DEWAX* OX Lines and the *dewax* Mutant

**FIGURE 3:**
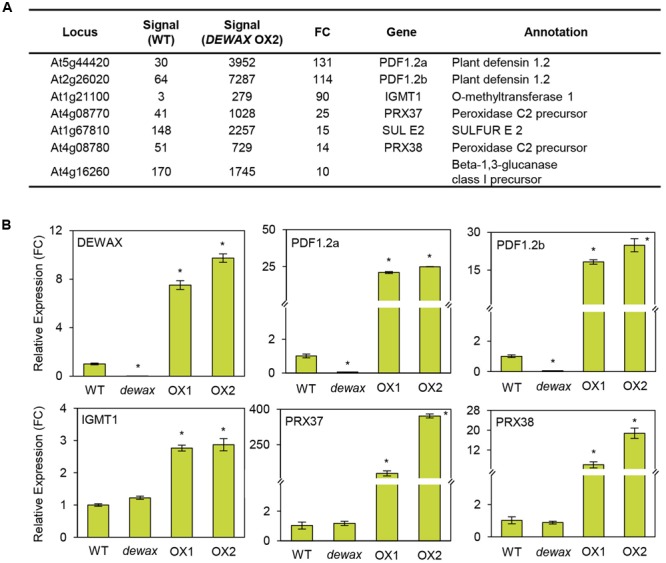
Microarray **(A)** and qRT-PCR **(B)** analyses of genes activated by *DEWAX* overexpression. **(A)** List of genes that were upregulated in stems of *DEWAX* OX2 relative to WT. Total RNA was isolated from stems of *DEWAX* OX2 and WT plants and subjected to microarray analysis using *Arabidopsis* ATH1 gene chips. FC, fold change. **(B)** Quantitative RT-PCR (qRT-PCR) analysis of genes upregulated in leaves of *DEWAX* OX2 as compared to WT. Total RNA was isolated from leaves of 4-week-old WT, *dewax*, and *DEWAX* overexpression lines (OX1 and OX2) and subjected to qRT-PCR analysis. The *PP2A* (At1g13320) gene was used as a reference to determine the RNA quality and quantity. Data were statistically analyzed using Student’s *t*-test (^∗^*P* < 0.01). Error bars indicate ± SD from triplicate experiments.

To obtain further insight in the DEWAX-mediated defense responses of *Arabidopsis*, comparative transcriptome analysis of WT and *DEWAX* OX was conducted (Go et al., 2014; E-MEXP-3781 at http://www.ebi.ac.uk/arrayexpress). Interestingly, the expression of genes involved in pathogen defense and ROS production was clearly upregulated in the stems of *DEWAX* OX as compared to those of WT (Penninckx et al., 2003; Almagro et al., 2009; Pedreira et al., 2011). Genes that were upregulated 10-fold and more in *DEWAX* OX2 than in WT are listed in **Figure 3A**. RT-PCR was carried out to confirm their transcript levels in 4-week-old WT, *dewax*, and *DEWAX* OX1 and OX2 leaves. The expression levels of *PDF1.2a* and *PDF1.2b*, involved in plant defense, were remarkably induced in *DEWAX* OX1 and OX2 leaves, but reduced in *dewax* as compared to WT leaves. The transcript levels of indole glucosinolate methyltransferase (*IGMT1*) and peroxidase C2 precursor (*PRX37* and *PRX38*) were increased in *DEWAX* OX1 and OX2 leaves, while no significant downregulation was observed in *dewax* relative to WT. No noticeable differences in the expression of *At1g67810* and *At4g16260*, encoding *SULFUR* (Stimulate CpNifS Cysteine Desulfurase Activity) and a β-1,3-glucanase precursor, respectively, were observed in *dewax*, and *DEWAX* OX1 and OX2 leaves (**Figure 3B** and Supplementary Figure S1).

### DEWAX Functions as a Transcriptional Activator

**FIGURE 4:**
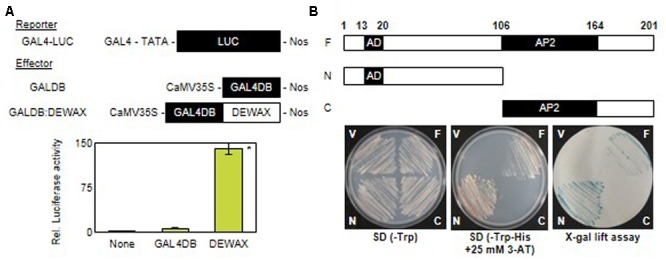
Transcriptional activation assay of DEWAX gene in tobacco protoplast and yeast. **(A)** Relative luciferase activities in tobacco protoplasts co-transfected with reporter and effector plasmids. The luciferase activities are expressed relative to values obtained with the reporter construct alone (none). GAL4, galactose-inducible gene promoter; LUC, luciferase; GAL4DB, GAL4 DNA-binding domain; Nos, nopaline synthase terminator; CaMV35, cauliflower mosaic virus 35S promoter. Biological triplicates were averaged and statistically analyzed using Student’s *t*-test (^∗^*P* < 0.01). Error bars indicate ± SD of the means. **(B)** Growth and β-galactosidase (X-Gal) assay of yeast transformed with F, N, or C construct on SD/Trp^-^ and SD-His^-^ plus 25 mM 3-AT media; F, full-length of the *DEWAX* present in pGBKT7; N, N-terminal domain of *DEWAX* present in pGBKT7; C, C-terminal domain of *DEWAX* present in pGBKT7; V, pGBKT7 plasmid without *DEWAX* as a control; AD, activation domain; AP, AP2 domain of the *DEWAX* required for DNA binding.

DEWAX has been reported to function as a transcriptional repressor in cuticular wax biosynthesis (Go et al., 2014). However, upregulation of defense-related genes in *DEWAX* OX and their downregulation in *dewax* prompted us to examine whether DEWAX acts as a transcriptional activator in tobacco protoplasts and yeast cells. A reporter construct, GAL4-LUC, harbors a luciferase gene driven by a promoter including a GAL4 motif. *DEWA*X was translationally ligated to the C-terminus of the GAL4 DNA-binding domain under the control of CaMV35S promoter in pBI221 vector (Lee et al., 2013) and used as an effector construct, GAL4DB:DEWAX. Tobacco protoplasts were transformed with a GAL4-LUC reporter construct only or co-transformed with a GAL4-LUC construct and an effector construct, GAL4DB or GAL4DB:DEWAX. Strong luciferase activities were observed in protoplasts transformed with GAL4-LUC reporter and a GAL4DB:DEWAX effector constructs as compared to those transformed with GAL4-LUC reporter and GAL4DB effector constructs (**Figure 4A**).

In addition, the full-length (F, amino acids 1 to 201), N-terminal (N, amino acids 1 to 106), and C-terminal (C, amino acids 107 to 201) regions of *DEWAX* were cloned downstream of the GAL4-binding domain in the pGBKT7 vector. Yeast Y190 cells, which possess the *HIS3* and *lacZ* genes driven by the promoter including GAL4-responsive elements, were transformed with the constructed vectors. Yeast cells that were able to survived on tryptophan-deficient selective medium (SD/-Trp) were further selected on tryptophan*-* and histidine-deficient medium (SD/-Trp-His) supplemented with 10 mM 3-amino-1, 2, 4-aminotriazole (3-AT) and subsequently subjected to an X-gal filter-lifting assay. Yeast cells transformed with the F or N construct survived on SD/-Trp-His medium supplemented with 10 mM 3-AT, and generated blue colonies by the β-galactosidase activities in X-gal lift assay. However, yeast cells transformed with the pGBKT7 vector or the C construct did not survive on the selective medium, and showed no β-galactosidase activity. These results indicate that DEWAX is able to act as a transcriptional activator, and amino acid residues 1 to 106 of DEWAX are involved in transcriptional activation (**Figure 4B**).

### DEWAX Promotes the Expression of *PDF1.2 via* Direct Binding to Conserved Sequence Motifs in its Promoter

**FIGURE 5:**
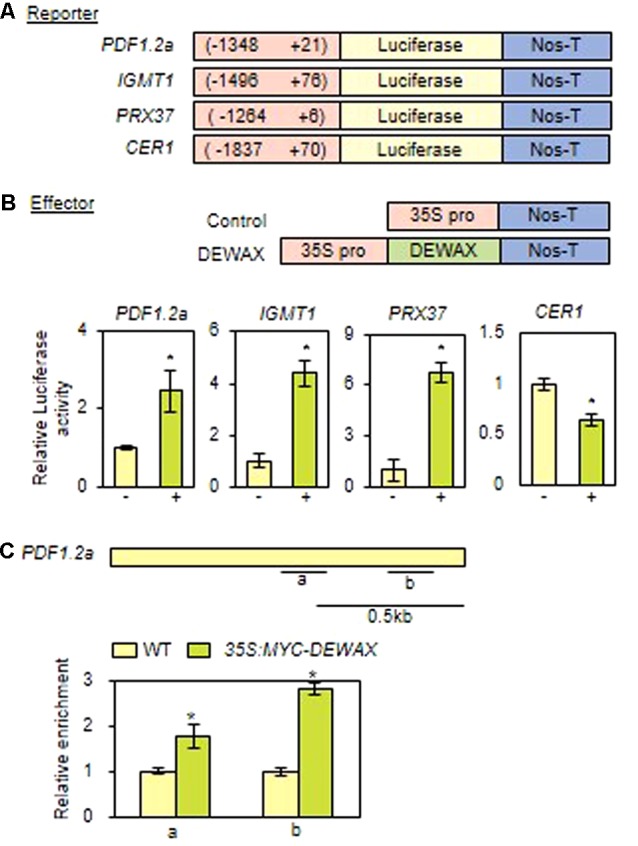
*DEWAX* upregulates the expression of the *PDF1.2* gene by direct binding to conserved sequence motifs in its gene promoter. **(A)** Schematic diagrams of reporter and effector constructs used for the transcriptional activation assay. In the reporter constructs, the promoter regions of *DEWAX*-activated genes were fused to the luciferase gene. In the effector constructs, *DEWAX* was cloned between the CaMV35S promoter and the terminator of the nopaline synthase gene (Nos-T). **(B)** Transcriptional activation assay of *DEWAX*-activated genes in tobacco protoplasts. The LUC activity was normalized to the GUS activity, which was from the internal control construct harboring the GUS gene. Mean fold change in relative luciferase activity was calculated by dividing the normalized luciferase activity obtained from the protoplasts transformed with the control effector construct. Data were statistically analyzed using Student’s *t*-test (^∗^*P* < 0.01). Error bars indicate ± SD of the mean from triplicate experiments. –, P35S; +, P35S-DEWAX. **(C)** Description of the promoter region of the *PDF1.2a* gene and chromatin immunoprecipitation (ChIP) assay. a and b indicate regions including consensus GCC-box motifs that were used in ChIP assay. In each qRT-PCR measurement, the value for WT was set to 1 after normalization against *actin7*. Data were statistically analyzed using Student’s *t*-test (^∗^*P* < 0.01). Error bars indicate ± SD of the mean from triplicate samples.

To examine whether DEWAX is able to activate the expression of the putative target genes, *PDF1.2a, IGMT1*, and *PRX37*, their promoter regions were transcriptionally ligated to the luciferase gene (*LUC*) in the Gal4-LUC binary vector, and used as reporter constructs (**Figure 5A**). *p35S* and *p35S-DEWAX* as effector constructs were used in transactivation assay of tobacco protoplasts (Go et al., 2014). After tobacco protoplasts were co-transformed with each reporter construct containing *LUC*, the effector construct, and the internal control construct harboring the *GUS* gene, luciferase (LUC) and β-glucuronidase (GUS) activities were measured, and the LUC activity was normalized to the GUS activity. When *DEWAX* was expressed, LUC activity was upregulated in the protoplasts transformed with *PDF1.2a, IGMT1*, and *PRX37* reporter constructs by approximately 2.3, 4.2, and 6.3-fold, respectively, but was downregulated in the protoplasts transformed with *CER1* by approximately twofold relative to the control co-transformed with *p35S* (**Figure 5B**).

We further investigated whether DEWAX directly interacts with the consensus GCC-box motifs of *PDF1.2a* promoter region using *35S:MYC-DEWAX* transgenic plants (Go et al., 2014). Quantitative real-time ChIP-PCR assay showed that DEWAX binds directly to the promoters of the *PDF1.2a* gene *in planta* (**Figure 5C**). This observation indicates that DEWAX activates the expression of *PDF1.2a* gene by direct interaction with its promoter region.

### Overexpression of *DEWAX* Enhanced the Resistance to *B. cinerea* in *C. sativa*

**FIGURE 6:**
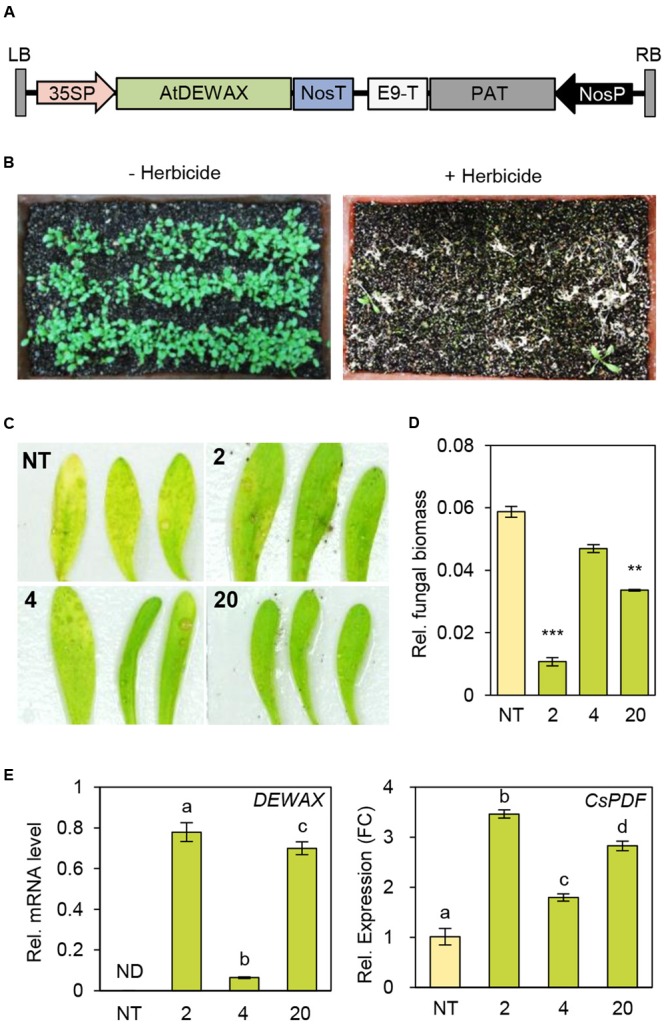
Generation of transgenic *Camelina sativa* plants overexpressing DEWAX. **(A)** Schematic diagram of the 35SP-*DEWAX* construct. LB, left border; 35SP, cauliflower mosaic virus 35S promoter; NosT, nopaline synthase terminator; E9-T, pea rbcs-E9 terminator; PAT, phosphinothricin acetyltransferase; NosP, nopaline synthase promoter; RB, right border. **(B)** Selection of transgenic *C. sativa* lines resistant to the herbicide BASTA. **(C)** Macroscopic symptoms on leaves of non-transgenic (NT) and *DEWAX C. sativa* OX plants (2, 4, and 20) at 4 days after inoculation with *B. cinerea*. **(D)** Quantification of *B. cinerea* biomass in NT and *DEWAX C. sativa* OX plants. The infected plants were collected at 4 days post inoculation, and genomic DNA was isolated. *B. cinerea* biomass was quantified by real-time quantitative PCR with *B. cinerea cutinase A*-specific primers and normalized to *C. sativa iASK* gene expression. All data represent the average of at least three plants. Error bars represent ± SD of the means. Data were statistically analyzed using Student’s *t*-test (^∗∗^*P* < 0.01, ^∗∗∗^*P* < 0.001). **(E)** qRT-PCR analysis of *DEWAX* and *C. sativa PDF* (*CsPDF*) genes. Total RNA was isolated from leaves of 5-week-old NT and *DEWAX C. sativa* OX plants and subjected to qRT-PCR analysis. The *CsActin11* gene (Hutcheon et al., 2010) was used as a reference to determine the RNA quality and quantity. Data were statistically analyzed using Student’s *t*-test (^∗∗^*P* < 0.01). Error bars indicate ± SD from triplicate experiments.

As *DEWAX* overexpression in *Arabidopsis* led to strong resistance to *B. cinerea*, we next evaluated how overexpression of *DEWAX* in a *Brassica* crop, *C. sativa*, affects disease resistance. The 35SP-*DEWAX* construct harboring *DEWAX* gene under the control of *CaMV 35S* promoter was used in the transformation of *C. sativa* (**Figure 6A**). Transgenic plants were screened through spraying of 0.03% BASTA herbicide (**Figure 6B**). Genomic DNA PCR analysis of non-transgenic (NT) and 16 transgenic plants (TO) using gene-specific primers (Supplementary Table S1) showed that *Arabidopsis DEWAX* was integrated in all transgenic plants tested (Supplementary Figure S2A). RT-PCR analysis revealed that the *DEWAX* gene was overexpressed in the leaves of all transgenic plants tested (Supplementary Figure S2B and Table S1).

Next, we examined the defense responses of NT and TO plants to *B. cinerea*. Visible disease symptoms, *B. cinerea* biomass, and the expression of *Arabidopsis DEWAX* were investigated in leaves of NT and TO-2, TO-4, TO-17, and TO-20 lines 4 days after inoculation with *B. cinerea*. Yellowing or chlorosis occurred more quickly in NT than in TO leaves (**Figure 6C**). To measure *B. cinerea* biomass, genomic DNA was isolated from the leaves 4 days after inoculation with *B. cinerea* and subjected to real-time quantitative PCR using *B. cinerea*-specific *cutinase A*-specific gene primers (Supplementary Table S1), while *C. sativa* GSK3/shaggy-like (*iASK*) gene was used for normalization. Compared to NT plants, all TO lines tested had lower *B. cinerea* biomass (**Figure 6D**). Subsequently quantitative RT-PCR analysis showed that the levels of *C. sativa PDF* increased by 2- to 3.5-fold in DEWAX OX lines compared with NT (**Figure 6E**). These results indicated that overexpression of *Arabidopsis DEWAX* confers tolerance to *B. cinerea* in transgenic *C. sativa*.

## Discussion

Plant immune responses are essential for not only growth and development, but also productivity and yields of crops. In this study, we investigated a plant defense mechanism related to the cuticle, which is the first defensive barrier of the aerial parts of plants. We revealed that overexpression of *DEWAX* increased cuticle permeability and ROS accumulation in *Arabidopsis*. DEWAX is a transcriptional activator that upregulates the expression of the defense-related genes, *PDF1.2a, IGMT1*, and *PRX37*, by binding to their gene promoters. In particular, DEWAX directly interacts with the GCC motifs in the *PDF1.2a* promoter. Overexpression of *DEWAX* conferred enhanced tolerance to necrotrophic fungal pathogen, *B. cinerea* in *Arabidopsis* and *C. sativa.* Therefore, convergent line of evidence supports that increased ROS levels as well as upregulation of defense-related genes might be related to the resistance to *B. cinerea* in *Arabidopsis* and *C. sativa* overexpressing *DEWAX*.

Go et al. (2014) reported the AP/ERF transcription factor DEWAX as a transcriptional repressor involved in cuticular wax biosynthesis during daily light/dark cycles. However, we revealed that DEWAX is also able to act as a transcriptional activator that induces defense-related genes in this study. AP2/ERF transcription factors are known to function as both negative and positive regulators (Ohta et al., 2001; Aharoni et al., 2004; Yant et al., 2010). The N-terminus of DEWAX harbors the “EDLL” motif, which is an acidic-type transcriptional activation domain of AP2/ERF transcription factors (Tiwari et al., 2012), which is consistent with our yeast transactivation assay results. Direct binding of DEWAX as well as binding of *OCTADECANOID-RESPONSIVE ARABIDOPSIS* (ORA)59 and ERF96 with the “EDLL” motif to the two GCC motifs in the *PDF1.2* promoter region, is essential for the expression of PDF1.2 (Solano et al., 1998; Zarei et al., 2011; Catinot et al., 2015). Although it remains unclear how exactly the bifunctional DEWAX regulates the expression of its target genes positively or negatively, possibly, DEWAX interacts with a co-activator, such as MED25 of the mediator complex, followed by the recruitment of transcriptional machinery including histone acetyltransferase, or with a co-repressor that interacts with histone deacetylase (Cevik et al., 2012; Huang and Geng, 2017).

The expression of the *PDF1.2a, PDF1.2b, IGMT1, PRX37*, and *PRX38* genes was upregulated in *Arabidopsis* overexpressing *DEWAX.* The *Arabidopsis* mutant *iop1* displaying induced expression of *PDF1.2* shows enhanced resistance against necrotrophic fungal pathogens, including *A. brassicicola, B. cinerea*, and *Plectosphaerella cucumerina* (Penninckx et al., 2003). Overexpression of wasabi defensin, a small and cysteine-rich protein, in rice, potato, and orchid, enhances their resistance to *B. cinerea* and *Magnaporthe grisea* (Lay and Anderson, 2005). In addition, mechanical wounding rapidly activates the expression of apoplastic class III peroxidase genes *PRX37* and *PRX38* and the release of peroxidases to the apoplast, where they generate ROS and oxidize phenolic substrates, resulting in increased cross-linking of the cell wall (Almagro et al., 2009; Pedreira et al., 2011; Minibayeva et al., 2015), suggesting that upregulation of *PRX37* and *PRX38* by DEWAX may be involved in elevating ROS accumulation in DEWAX OX lines. Moreover, IGMT1 catalyzes the transmethylation of 1-hydroxy-indol-3-yl-methyl glucosinolate intermediates and 4-hydroxy-indol-3-yl-methyl to 1-methoxy-indol-3-yl-methyl and 4-methoxyindol-3-ylmethylglucosinolate, respectively, which are activated by β-thioglucoside glucohydrolase for antifungal defense (Bednarek et al., 2009; Clay et al., 2009). Therefore, the upregulation of *PDF1.2, PRX37, PRX38*, and IGMT1 by DEWAX might be important for plant innate immunity.

Some *Arabidopsis* mutants with increased cuticular permeability exhibit resistance to pathogen infection (Nawrath, 2006; Serrano et al., 2014). For example, *lcr/lacerate/cyp86A8, bdg, lacs-2* and *-3, fdh, fec1*, and *myb96* showed enhanced resistance to *B. cinerea*, similar to our observations in *DEWAX* OX lines (Yephremov et al., 1999; Wellesen et al., 2001; Bessire et al., 2007, 2011; Chassot et al., 2007; Voisin et al., 2009; Seo et al., 2011). We also observed increased hydrogen peroxide levels in *DEWAX* OX leaves (**Figure 1B**), which is consistent with the results in the *Arabidopsis* mutants, *bdg* and *lacs2* (L’Haridon et al., 2011), although we could not exclude the possibility that the higher levels of hydrogen peroxide in *DEWAX* OX leaves may be attributed, to some extent, to smaller *DEWAX* OX leaves. Transgenic lines overexpressing oxalate decarboxylase and thus, having higher levels of oxalic acid, which inhibits ROS production, showed decreased innate immune response (Cessna et al., 2000; L’Haridon et al., 2011). Therefore, elevated ROS production in *Arabidopsis* overexpressing *DEWAX* might be involved in the resistance to *B. cinerea*, although the molecular mechanisms underlying immune responses of mutants with permeable cuticle to *B. cinerea* are still largely unknown.

The expression of *PDF1.2* was significantly upregulated in *Arabidopsis* seedlings treated with jasmonic acid (JA) and ethephon, a form of liquid ethylene (Lorenzo et al., 2003; Pre et al., 2008). The JA- and ethylene-responsive expression of *PDF1.2* depended on ERF1 and ORA59 transcription factors, which act as integrators of JA and ethylene signaling pathways. Overexpression of *ERF1* and *ORA59* caused increased resistance, and *ora59* mutant enhanced susceptibility to *B. cinerea* (Berrocal-Lobo et al., 2002; Pre et al., 2008). However, the transcript levels of *DEWAX* were elevated neither by JA nor by ethephon^1^, although *DEWAX* is classified with *ERF1* and *ORA59* into the group IX of *Arabidopsis AP2/ERF* gene family (Nakano et al., 2006). Based on the previous reports that *Arabidopsis* defense responses to herbivores and pathogens are regulated by circadian rhythms (Wang et al., 2011; Goodspeed et al., 2012), it would be interesting to examine whether upregulation of defense-related genes, including *PDF1.2* by the diurnally controlled DEWAX may be important in circadian clock-mediated plant innate immunity. In addition, JA and salicylic acid are known to play a role as an antagonist in plant innate immunity (Thaler et al., 2012). The induction of the *PDF1.2* gene in *DEWAX*-overexpressing *Arabidopsis* indicates that JA-associated defense responses are activated, and this may cause the suppression of SA-related signaling. This hypothesis supports that *DEWAX*-overexpressing plants are more resistant to the necrotropic fungal pathogen, *B. cinerea*, but are more susceptible to the semi-biotropic bacterial pathogen, *Pto DC3000* compared with WT.

Although *C. sativa* is considered to be resistant to many diseases, it is reportedly sensitive to some fungal and bacterial pathogens causing wilting, root rot, clubroot, white rust, and downy mildew diseases (Séguin-Swartz et al., 2009; Zakharchenko et al., 2013). Zakharchenko et al. (2013) reported that transgenic *C. sativa* overexpressing the antimicrobial peptide cecropin P1 exhibits enhanced resistance to *Erwinia carotovora* and *Fusarium sporotrichioides*. In this study, the *Arabidopsis dewax* mutant showed increased resistance to *Pto* DC3000, whereas overexpression of *DEWAX* conferred elevated resistance to *B. cinerea* in *Arabidopsis* and *C. sativa*, suggesting the potential for developing *Brassica* lines resistant to various diseases. In conclusion, our study revealed that DEWAX is a transcriptional activator that upregulates the expression of defense-related genes, and overexpression of DEWAX enhances the resistance to *B. cinerea* in *Arabidopsis* and *C. sativa*. We provide that DEWAX-mediated defense mechanisms may be applicable in the improvement of crops with enhanced resistance to fungal and bacterial pathogens.

## Author Contributions

SJ, YG, and MS conceived and designed research. SJ, YG, and HC conducted experiments. SJ, JP, and MS analyzed data. SJ, JP, and MS wrote the manuscript. All authors read and approved the manuscript.

## Conflict of Interest Statement

The authors declare that the research was conducted in the absence of any commercial or financial relationships that could be construed as a potential conflict of interest.

## Footnotes

1 http://bar.utoronto.ca/efp/cgi-bin/efpWeb.cgi
