# Supplementary material for: DEWAX Transcription Factor Is Involved in Resistance to Botrytis cinerea in Arabidopsis thaliana and Camelina sativa
Source: Front Plant Sci. 2017 Jul 11;8:1210. doi: 10.3389/fpls.2017.01210 (PMC5504226; doi:10.3389/fpls.2017.01210)
Supplement: Supplementary file 1 [file Table_1.PDF]

Supplemental Table 1. Oligonucleotide sequences used in this study

Reaction	Primer name	Sequence information
Transcriptional activation assay	PDF1.2A pro F1	5'-GGTCGACGTACGACGTTGGACTGTTTC
	PDF1.2A pro R1	5'-AACTAGTGTATAGATGTATGTGTTGTG
	IGMT1 pro F1	5'-GGTCGACCAAGATTTGACGAGTCACAAG
	IGMT1 pro R1	5'-AACTAGTGGCACTCTACGTGATCACAC
	Peroxi37 pro F1	5'-GGTCGACCTCACATATTACAGCAGCCC
	Peroxi37 pro R1	5'-AACTAGTGGCCAAGGTTCTAGCTTGGC
Transactivation assay in yeast	At5g61590NF1	5’-CCCCGGGAATGGAGACTTTTGAGGAAAGC
	At5g61590NR1	5’-GGTCGACCGTTTCTTCAAACCTCTTTG
	At5g61590CF1	5’-CCCCGGGAGAAACGAGACATTACAGAGGC
	At5g61590CR1	5’-GGTCGACGTTTGATGACGATGATGAAG
ChIP assay	ACT2-ChIP_F	5'-CGTTTCGCTTTCCTTAGTGTTAGCT
	ACT2-ChIP_R	5'-AGCGAACGGATCTAGAGACTCACCTTG
	PDF1.2 CHIP F1	5'-CCCTGAGCCTCTCACTTGCGG
	PDF1.2 CHIP R1	5'-GAACGGAGAGGGCAGGAACC
	PDF1.2 CHIP F2	5'-CCAGCCGCCCATGTGAACG
	PDF1.2 CHIP R2	5'-GTTGATGGCTGGTTTCTCC
RT-PCR	EIF4A1-F	5'-CCGTGGTTTCAAGGACCAGATC
	EIF4A1-R	5'-GTCTGTGAGCCAATCAACCTTAC
	PDF1.2A real-F1	5'-GGTGGAAGCACAGAAGTTGT
	PDF1.2A real-R1	5'-AATACACACGATTTAGCACC
	IGMT1 real-F1	5'-GTTCTGGTGGAAAAGAGCGATC
	IGMT1 real-R1	5'-CAGAATTCAATAATCCAGCAG
	PRX37 real-F1	5'-GAGCGACCAAGAGTTATTCTC
	PRX37 real-R1	5'-CTACTCATCCTTATCATTGCC
	SUL E2-real-F1	5'-CGGCAAGGAACAGTCAAGGG
	SUL E2-real-R1	5'-GATCATGTTGGTGCGGTGGTC
	PRX38-real-F1	5'-GTTCGAGCATACGCTGATGG
	PRX38-real-R1	5'-CACCACTCTACAGTTCAATC
	4g16260 real-F1	5'-GAGAGAGAACAGAGGAACAC
	4g16260 real-R1	5'-CAACCGCCGTACCGTCTCTC
Genomic DNA PCR (Camelina)	CaMV35S-F1	5'-GCCTCTGCCGACAGTGGTCCCAAAG
	At5g61590CR1	5'-GGTCGACGTTTGATGACGATGATGAAG
mRNA expression analysis (Camelina)	At5g61590CF1	5'-CCCCGGGAGAAACGAGACATTACAGAGGC
	Nos-transcripts-R	5'-TTGAACGATCGGGGAAATTC
	CsPDF-F1	5'-CACACAACACATCCATTGAAAAC
	CsPDF-R2	5'-TAACACATGTGAGCTGGGAATA
	CsACT11-F	5'-ACAATTTCCCGCTCTGCTGTTGTG
	CsACT11-R	5'-AGGGTTTCTCTCTTCCACATGCCA
