# Supplementary figures and images for: DEWAX Transcription Factor Is Involved in Resistance to Botrytis cinerea in Arabidopsis thaliana and Camelina sativa
Source: Front Plant Sci. 2017 Jul 11;8:1210. doi: 10.3389/fpls.2017.01210 (PMC5504226; doi:10.3389/fpls.2017.01210)

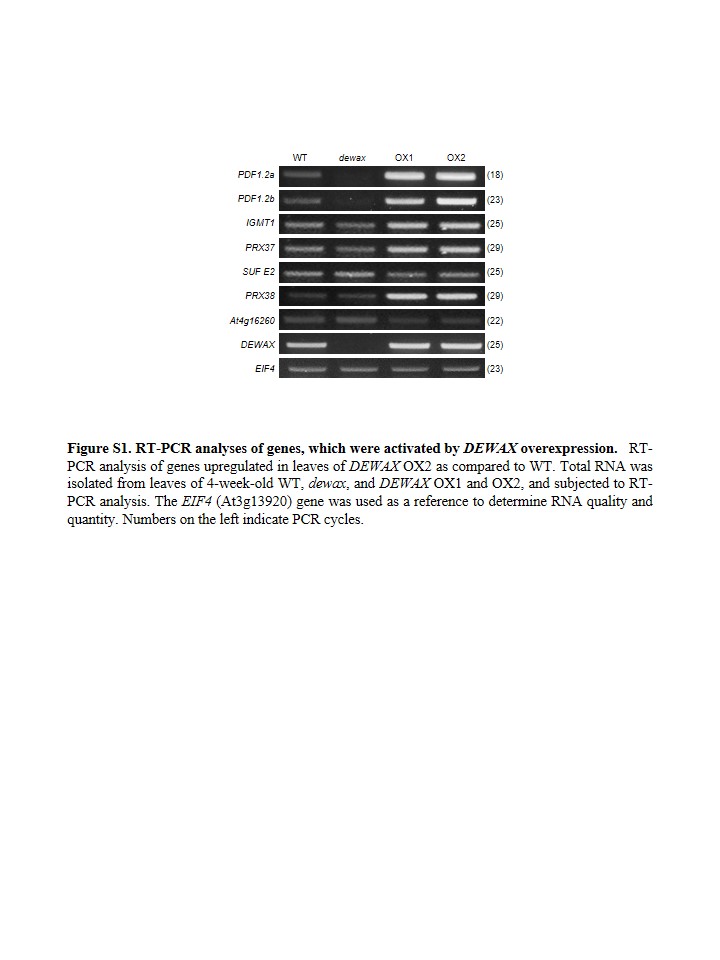

Supplement: Supplementary file 2 [file Image_1.JPEG]

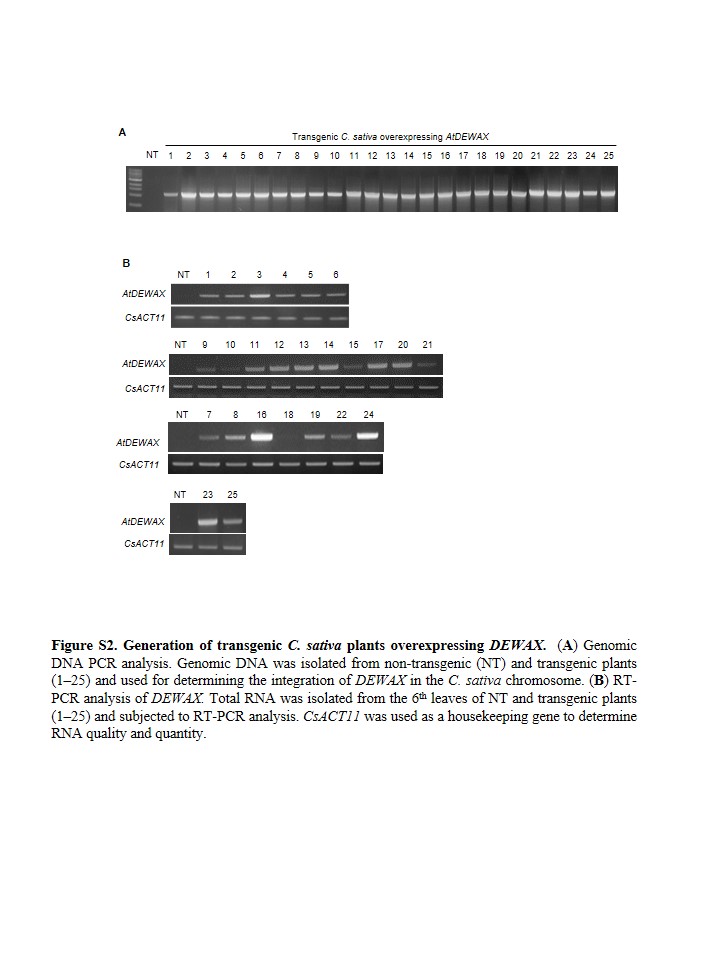

Supplement: Supplementary file 3 [file Image_2.JPEG]
